# pGVG: a new Gateway-compatible vector for transformation of sugarcane and other monocot crops

**DOI:** 10.1590/1678-4685-GMB-2017-0262

**Published:** 2018-06-11

**Authors:** Giovanna V. Guidelli, Lucia Mattiello, Rafael H. Gallinari, Paulo Cezar de Lucca, Marcelo Menossi

**Affiliations:** 1 Universidade Estadual de Campinas Universidade Estadual de Campinas Instituto de Biologia Departamento de Genética CampinasSP Brazil Laboratório de Genoma Funcional, Universidade Estadual de Campinas, Departamento de Genética, Evolução e Bioagentes, Instituto de Biologia, Campinas, SP, Brazil; 2 Universidade Estadual de Campinas Universidade Estadual de Campinas Instituto de Biologia Departamento de Genética, Evolução, Microbiologia e Imunologia CampinasSP Brazil PangeiaBiotech, Universidade Estadual de Campinas, Departamento de Genética, Evolução, Microbiologia e Imunologia, Instituto de Biologia, Campinas, SP, Brazil

**Keywords:** Monocots, sugarcane, vector, Gateway technology, genetic transformation

## Abstract

The successful development of genetically engineered monocots using *Agrobacterium-*mediated transformation has created an increasing demand for compatible vectors. We have developed a new expression vector, pGVG, for efficient transformation and expression of different constructs for gene overexpression and silencing in sugarcane. The pCAMBIA2300 binary vector was modified by adding Gateway recombination sites for fast gene transfer between vectors and the maize polyubiquitin promoter Ubi-1 (*ZmUbi1*), which is known to drive high gene expression levels in monocots. Transformation efficiency using the pGVG vector reached up to 14 transgenic events per gram of transformed callus. Transgenic plants expressing the β-glucuronidase (*GUS*) reporter gene from pGVG showed high levels of GUS activity. qRT-PCR evaluations demonstrated success for both overexpression and hairpin-based silencing cassettes. Therefore, pGVG is suitable for plant transformation and subsequent applications for high-throughput production of stable transgenic sugarcane. The use of an expression cassette based on the *ZmUbi1* promoter opens the possibility of using pGVG in other monocot species.

Sugarcane (*Saccharum spp* L.) is one of the most economically important crops due to its bioenergetic potential and is recognized as a source of renewable energy (Gianotto *et al.*, 2011). Genetic transformation methods are powerful biotechnological tools to improve yield and *Agrobacterium*-mediated transformation, initially restricted to dicots, has been successfully used in many monocot plants (Gelvin, 2003; Shrawat and Lörz, 2006; Hiei *et al.*, 2014; Slamet-Loedin *et al.*, 2014; Mayavan *et al.*, 2015). This method became one of the main approaches used to produce transgenic plants due to its simplicity, low-cost equipment needs and delivery of one or few copies of larger gene insertions. Furthermore the transfer DNA has greater stability, favoring its heritability in comparison to other transformation methods (Elliott *et al.*, 1998; Hansen and Wright, 1999; Travella *et al.*, 2005). Despite that, the availability of vector system compatible for monocots is limited, and most expression vectors are based on the CaMV 35S promoter, which generates lower expression levels in monocots (Mann *et al.*, 2012). There are other vectors for monocot transformation, however they are limited to gene silencing (Karimi *et al.*, 2002), lack epitope tags for protein detection/isolation (Mann *et al.*, 2012), present *ZmUbi1* promoter driving both gene of interest and selection cassettes, which can cause gene silencing (De Wilde *et al.*, 2000; Butaye *et al.*, 2005; Himmelbach *et al.*, 2007; Mann *et al.*, 2012) or show regeneration problems due to the use of hygromycin selection (Joyce *et al.*, 2010).

In this study, we describe the construction and functional validation of a vector (pGVG) for gene functional analysis in sugarcane and other monocots. The pGVG vector (Figure 1) is based on the backbone from pCAMBIA2300 binary vector (CAMBIA, Canberra, Australia) that possesses the *NPTII* gene as selection marker. The latter is one of the most efficient markers for transgenic sugarcane callus selection and certified for use in commercial transgenic species (Zhangsun *et al.*, 2007; Joyce *et al.*, 2010). The pGVG vector presents a Gateway cassette (*att*R1-Cm^r^-*ccd*B-*att*R2) under control of the *ZmUbi1* promoter and CaMV 35S terminator for gene overexpression or silencing. By incorporating the Gateway cloning technology, pGVG allows a fast and easy exchange of DNA fragments between vectors, without using restriction endonucleases and ligases from traditional cloning. Target DNA flanked by *att*L recombination sites is easily transferred to *att*R site-compatible destination vectors using the LR clonase enzyme. In this process, the lethal *ccd*B gene is moved from the destination plasmid to the entry vector, facilitating the selection of recombinant constructions (Katzen, 2007). Entry vectors such as pCR8GW TOPO (Invitrogen, Life Technologies, USA), with resistance to spectinomycin, are suitable for direct recombination with pGVG. In cases where both entry and pGVG destination vectors have the same bacterial selectable marker it is indicated to use the PCR product flanked by the recombination sites to assure high efficiency of recombination. The *ZmUbi1* promoter was cloned in pGVG with the 5’ untranslated region and the first intron of the *Ubi-1* gene, which is associated with enhanced transgene expression in monocot (Callis *et al.*, 1988; Bruce and Quail, 1990; McElroy *et al.*, 1990; Vasil *et al.*, 1993; Christensen and Quail, 1996). This promoter allows high levels of gene expression or RNAi-mediated suppression in most tissue types during most stages of plant development (Cornejo *et al.*, 1993; Mann *et al.*, 2012), and is used to produce stable transgenic monocot plants (Gallo-Meagher and Irvine, 1996; Ma *et al.*, 2000; Kinkema *et al.*, 2014). Additionally, pGVG presents a FLAG-tag sequence (DYKDDDDK) inserted upstream of the CaMV 35S terminator for C-terminal fusion with the target protein.

**Figure 1:**
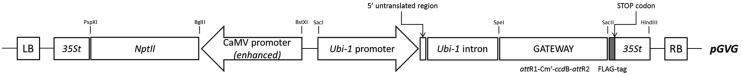
Schematic structure of the pGVG vector. This vector contains the backbone from pCAMBIA2300, modified by the insertion of the *ZmUbi1* promoter (including 5’ untranslated exon and first intron) for strong transgene overexpression and the CaMV 35S terminator. The sequences from the Gateway system were inserted between the *ZmUBi1* promoter and the CaMV 35S terminator. A FLAG-tag was positioned upstream the terminator to facilitate target protein isolation. The vector also contains the *NPTII* gene as plant selectable marker under control of the enhanced CaMV 35S promoter. Cm^r^: chloramphenicol-resistance gene. *ccd*B: lethal gene. RB: right border. LB: left border.

To produce transgenic lines, sugarcane plants (SP80-3280) were cultivated in greenhouse (IAC, Ribeirão Preto, Brazil) for six months, and the meristematic region from shoot apex was used to generate explants. This material was cultivated in MS maintenance medium [4.33 g/L MS salts (Murashige and Skoog, 1962), 1 mL/L MS vitamins, 0.15 g/L citric acid, 0.5 g/L casein hydrolysate, 25 g/L sucrose, 12 g/L mannitol, 100 mg/L proline, 3 mg/L 2-4 dichlorophenoxyacetic acid (2,4-D) and 2.8 g/L phytagel] at 26 °C in the dark, until the generation of embryogenic calli. Several constructs based on pGVG (see below) were transferred to *Agrobacterium tumefaciens* (EHA105 strain) by heat shock. Bacterial cultures were incubated with sugarcane calli under vacuum pressure for 5 min and transferred to co-cultivation medium (4.33 g/L MS salts, 1 mL/L MS vitamins, 3 mg/L 2,4-D, 0.15 g/L citric acid, 25 g/L sucrose and 3.5 g/L phytagel) at 22 °C, in the dark for 3 days. Subsequently, the calli were kept in resting medium (4.33 g/L MS salts, 1 mL/L MS vitamins, 3 mg/L 2,4-D, 0.5 g/L casein hydrolysate, 0.15 g/L citric acid, 25 g/L sucrose, 100 mg/L proline, 2.8 g/L phytagel and 200 mg/mL timentin) at 26 °C, in the dark for 6 days. Following the resting phase, the transformed calli were transferred to a selective regeneration medium [4.33 g/L MS salts, 1 mL/L MS vitamins, 25 g/L sucrose, 5 mg/mL CuSO_4_, 1 mg/mL benzylaminopurine (BAP), 7 g/L agar, 200 mg/mL timentin and 40 mg/L geneticin] at 26 °C, during 14 days with 16 h photoperiod. The transgenic events were kept in medium without phytohormones (4.33 g/L MS salts, 1 mL/L MS vitamins, 25 g/L sucrose, 7 g/L agar, 200 mg/mL timentin and 40 mg/L geneticin) to induce growth and rooting. Plants transformed with pGVG empty vector and wild-type plants were used as negative controls.

The functionality of pGVG was evaluated using the *GUS* reporter gene. The coding sequence of *GUS* gene was amplified from the construction pENTR -gus (Invitrogen, Life Technologies, Carlsbad, CA), using specific primers designed in the *att*L Gateway recombination sites. The purified PCR product was recombined with pGVG using Gateway® LR Clonase® II enzyme (Invitrogen, Life Technologies). The resulting vector was introduced into *Agrobacterium* and used for calli transformation. Transgene expression was assessed by GUS histochemical staining (Jefferson, 1987). Strong GUS activity was detected in callus and whole plants (Figure 2), confirming that pGVG is suitable for sugarcane transformation.

**Figure 2:**
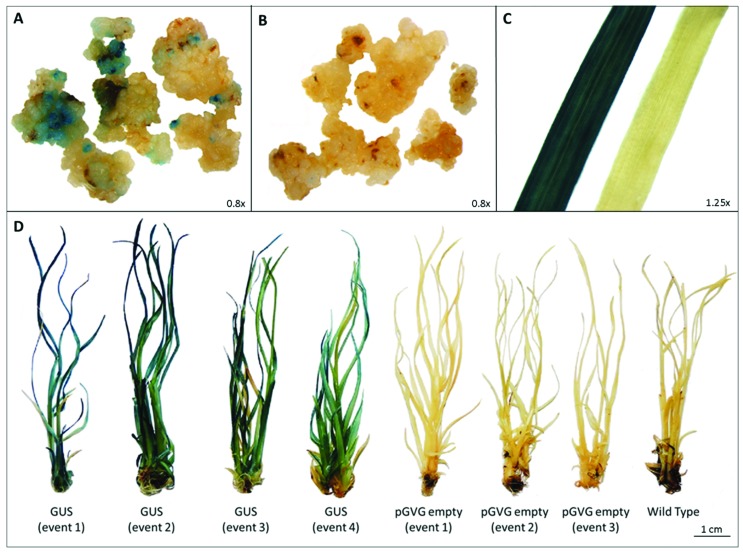
GUS expression on transgenic sugarcane tissues obtained from Agrobacterium-mediated transformation system using the pGVG vector. Transformed (A) and untransformed (B) calli, three weeks after the co-culture period; (C) leaves from a transformed (left) and untransformed (right) plant; (D) transformed (GUS, pGVG empty) and untransformed (Wild Type) plants, 4 months after the co-culture period.

To further evaluate the transformation capacity of pGVG, sugarcane genes related with different biological processes were tested using overexpression and RNAi-mediated silencing constructs (Table 1). The vector was able to transform sugarcane plants with genes of different sizes for both construct types. The overall transformation efficiency showed variation, probably reflecting differences in callus quality, culture medium, age and selective subculturing, which affect both transformation and plant regeneration (Pacurar *et al.*, 2008; Basnayake *et al.*, 2011).

**Table 1:** Transformation efficiency in sugarcane using pGVG.

Gene function	Type of cassette^a^	Events	Callus (g)	Efficiency^b^	Construct size (bp)
Gene 1 - Drought stress	OE	153	15	10.20	573
Gene 2 - Drought stress	OE	215	15	14.33	1,185
Gene 3 - Drought stress	OE	142	15	9.46	384
Gene 4 - Drought stress	OE	71	15	4.73	453
Gene 5 - Drought stress	OE	85	10	8.50	243
Gene 6 - Drought stress	OE	59	10	5.90	849
Gene 7 - Drought stress	OE	125	10	12.50	942
Gene 8 - Drought stress	OE	89	10	8.90	609
Gene 9 - Growth	OE	75	10	7.50	1,086
Genes 9, 10 and 11 - Growth	HS	71	10	7.10	892
Gene 9 - Growth	HS	84	10	8.40	632
Gene 12 - Cell wall biosynthesis	HS	42	15	2.80	1,125
Gene 13 - Cell wall biosynthesis	HS	129	15	8.60	923
Gene 14 - Cell wall biosynthesis	HS	182	15	12.13	605
Genes 15, 16 and 17 - Development	HS	24	15	1.60	2,795
Genes 15 and 16 - Development	HS	80	10	8.00	1,314
pGVG	Empty vector	30	7	4.29	1,455
pGVG	Empty vector	6	5	1.20	1,455
pGVG	Empty vector	15	7	2.14	1,455

a OE: overexpression, HS: hairpin silencing;

b Transformation efficiency expressed as the number of transgenic plants per gram of fresh callus matter.

Analyses of gene expression were performed through qRT-PCR using gene-specific primers and the polyubiquitin gene (SCCCST2001G02.g) as internal control for normalization (Papini-Terzi *et al.*, 2005) (Figure 3). The results demonstrated that sugarcane genes 1 and 2 were up-regulated in different levels in the transgenic lines when compared with endogenous levels observed in the empty vector control (Figure 3A,B). Additionally, a unique hairpin construction that targets three genes of the same family (genes 15, 16 and 17) silenced each member reaching up to 92% of down-regulation (Figure 3C). These data show that pGVG is able to produce efficient transgene overexpression and suppression of target genes.

**Figure 3:**
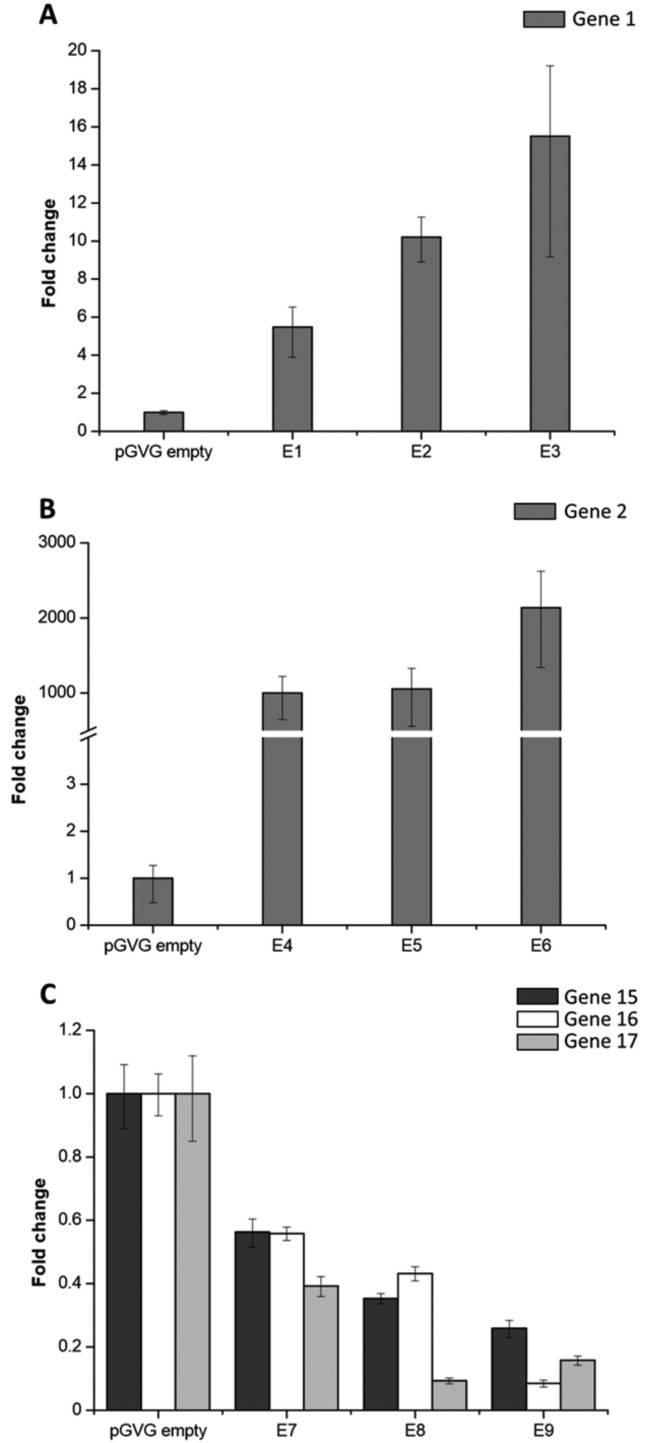
Expression levels of different sugarcane genes induced or repressed in transgenic sugarcane plants using the pGVG vector. Leaves from transgenic plants were used to extract RNA and the transcripts were quantified using RT-qPCR (A) Overexpression of gene 1 (drought stress-related) in three independent lines (E1, E2, and E3) compared with control (pGVG empty). (B) Overexpression of gene 2 (drought stress-related) in three independent lines (E4, E5 and E6) compared with control (pGVG empty). (C) RNAi-mediated suppression of the genes 15, 16 and 17 (development related; triple silencing) in three independent lines (E7, E8 and E9) compared with control (pGVG empty). Data represent the mean of three biological replicates. Bars indicate the standard error. The expression data refer to the transgene and the correspondent endogenous gene levels. The genes named here are the same as described in Table 1.

Therefore, the combination of adequate plant selectable markers, Gateway technology, and stable and strong promoters in the pGVG vector assures effective transformation and plant regeneration demonstrated by *GUS* reporter gene expression and qRT-PCR assays. This vector can be used in overexpression and RNAi-mediated silencing of sequences of interest in sugarcane plants that will greatly facilitate the functional characterization of genes. All characteristics incorporated into pGVG certainly will allow it to be used successful in several other monocot species.
